# Visualization of α-synuclein trafficking via nanogold labeling and electron microscopy

**DOI:** 10.1016/j.xpro.2023.102113

**Published:** 2023-02-24

**Authors:** Armin Bayati, Wen Luo, Esther Del Cid-Pellitero, Edward A. Fon, Thomas M. Durcan, Peter S. McPherson

**Affiliations:** 1 Department of Neurology and Neurosurgery, Montreal Neurological Institute, McGill University, Montreal, QC H3A 2B4, Canada; 2 The Neuro’s Early Drug Discovery Unit (EDDU), McGill University, Montreal, QC H3A 2B4, Canada

**Keywords:** Cell culture, Microscopy, Neuroscience, Molecular/Chemical Probes

## Abstract

There is conflicting evidence regarding the mechanisms of α-synuclein internalization, and its trafficking itinerary following cellular entry remains largely unknown. To examine these issues, we describe steps for coupling α-synuclein preformed fibrils (PFFs) to nanogold beads and their subsequent characterization by electron microscopy (EM). Then we describe the uptake of conjugated PFFs by U2OS cells plated on Permanox 8-well chamber slides. This process eliminates the reliance on antibody specificity and the need to employ complex immunoEM staining protocols.

For complete details on the use and execution of this protocol, please refer to Bayati et al. (2022).^1^

## Highlights

• Conjugation of α-synuclein preformed fibrils (PFFs) with nanogold beads
• Characterization and optimization of PFF conjugation
• Cell culture preparation for EM imaging and following PFF internalization

## Abstract (editor highlights)

Publisher’s note: Undertaking any experimental protocol requires adherence to local institutional guidelines for laboratory safety and ethics.

## Graphical abstract

**Figure:**
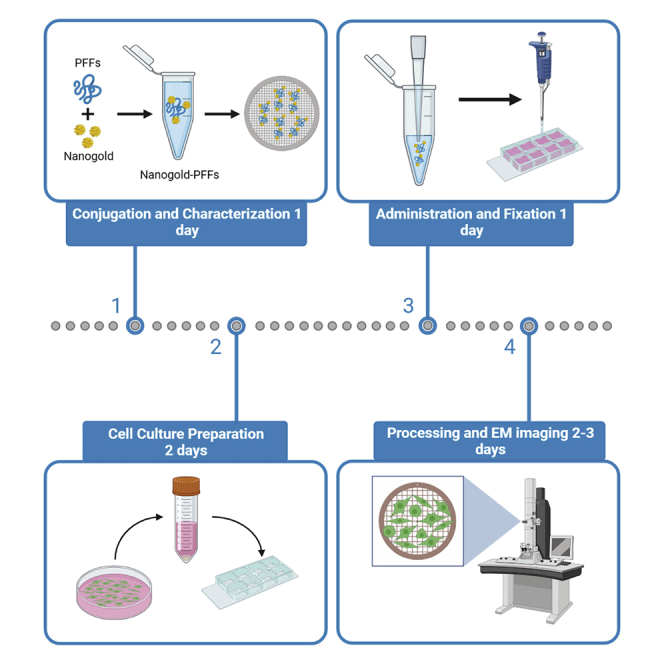

## Before you begin

The protocol below outlines the conjugation of PFFs with nanogold beads, to observe PFFs internalization using EM. Before beginning this conjugation process, please plan to have the necessary protocol and reagents in place to produce α-synuclein PFFs. We recommend following the production protocol established by Maneca et al.^2^ Below we outline a summary of the production process.

### Production of α-synuclein preformed fibrils (PFFs)

**Timing: ∼12 d**

1. Produce recombinant human α-synuclein monomers.
  a. Transform the pGEX6 plasmid construct into BL21 (DE3) *E. coli* for expression. The GST tag is incorporated on the N terminus of α-synuclein along with a 3C protease cleavage site providing a straightforward route to enrich and cleave GST-α-synuclein using glutathione beads. Through this route, high levels of monomeric synuclein can be enriched and purified from GST-α-synuclein.
  b. Separately, express GST-α-synuclein and GST-HRV 3C protease in cultures using Isopropyl-β-D-thiogalactoside (IPTG) induction and purify them with a Glutathione Sepharose 4B affinity column.
  c. Cleave off the GST tag from GST-α-synuclein with GST-HRV 3C protease.
  d. Remove GST and GST-HRV 3C protease with the Glutathione Sepharose 4B column and purify untagged α-synuclein to homogeneity as demonstrated by SDS-PAGE gel (with purity at least >95%).
  e. Exchange the purified α-synuclein into PBS buffer, pH 7.4, and adjust the final protein concentration to 5 mg/mL as determined by Bradford assay with Bovine Serum Albumin (BSA) as a reference.
  f. Filter-sterilize α-synuclein solution through a 0.22-μm filter. Make 0.5 mL aliquots in each 1.5-mL low protein binding microtube and store at −80°C.
2. Generate α-synuclein PFFs.
  a. Place monomeric α-synuclein tube(s) in a thermomixer with constant shaking at 1,000 rpm at 37°C for 5 d. The contents in the tube(s) will become turbid/opaque, indicating the formation of aggregates.
  b. Sonicate the resulting PFFs in the Bioruptor Pico sonicator using at least 40 cycles of 30 s ON-30 s OFF program ensuring the particle size after sonication is ∼50–80 nm in hydrodynamic diameters as assessed using dynamic light scattering (DLS) Zetasizer Nano S (using the Malvern Zetasizer software).
  c. The sonicated PFFs should be stored in aliquots (5–50 μL depending on experimental needs) at −80°C.

***Note:*** The ideal concentration of PFFs required for conjugation can vary based on the size of the nanogold particles used (see the nanogold manufacturer’s instructions). For instance, for 5 nm nanogold beads, the manufacturer recommends a protein concentration of 5 mg/mL; conversely, with 10 nm nanogold beads, they recommend a 2.5 mg/mL concentration for the protein.

### Characterization of α-synuclein PFFs

**Timing: 2 days**

The following steps should be carried out on parafilm. This protocol is adapted from Del Cid Pellitero et al.^2^

**Figure 1:**
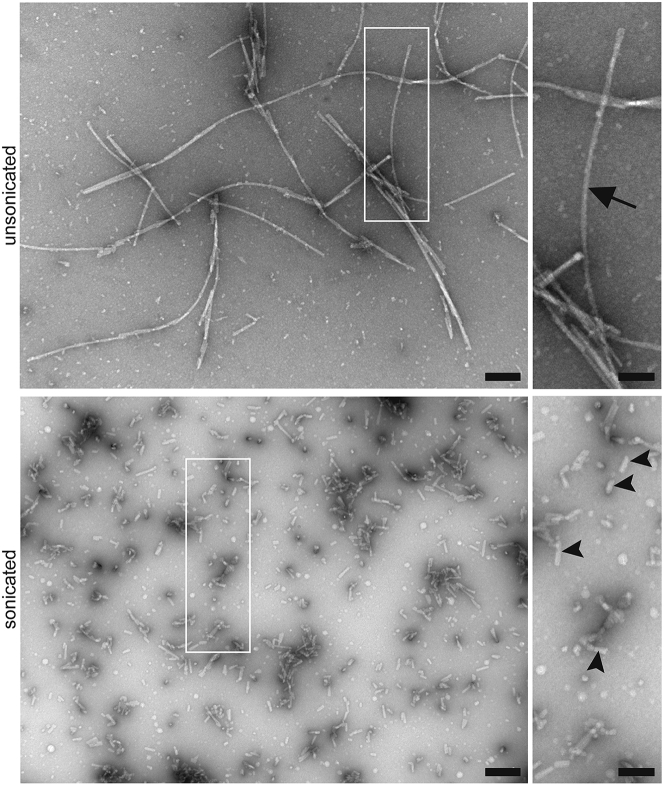
PFFs visualized using negative staining before and after sonication Samples are stained with uranyl acetate. The arrow points to an example of a long fibril. Arrowheads point to multiple examples of preformed fibrils with a length of less than 100 nm, Scale bar = 200 nm and 100 nm for insets.

3. Prepare a diluted sample of PFFs at 1 mg/mL.
  a. Dilute using ddH_2_O.
4. Place 5 μL of diluted PFFs on a carbon/formvar covered copper grid and allow the sample to sit for 10 min at room temperature.
  a. Using a Whatman filter paper, remove the liquid drop from the grid.
5. Fix PFFs on the grid using 2% PFA.
  a. Place one drop of 2% PFA onto the grid and let the sample sit for 10 min at 4°C.
  b. Remove the PFA drop with Whatman filter paper.
6. Wash the grid with ddH_2_O.
  a. Add one drop of ddH_2_O onto the grid for 1 min at room temperature.
  b. Remove the drop with Whatman filter paper.
  c. Repeat steps a-b once more.
7. Stain the grid with uranyl acetate.
  a. Centrifuge 1 mL of uranyl acetate at 14,000 × *g* for 1 min.
  b. Add one drop of the supernatant to the grid and wait for 5 min at room temperature.
8. Wash the grid with ddH_2_O.
  a. Repeat step 6.
9. Let the grid sit overnight on parafilm.
10. Visualize by EM to assess and measure the length of fibrils.
  a. See Figure 1 for example:

Characteristics of optimal PFFs:

• A mean length of less than 100 nm.
• Sonicated PFFs must retain the same thickness as unsonicated PFFs.

***Note:*** for PFFs visualization (unlabeled, use a magnification between ×10,000 and ×30,000).

***Note:*** The characterization of α-synuclein PFFs was adapted from Del Cid Pellitero et al.^2^

## Key resources table

**Table:** 

REAGENT or RESOURCE	SOURCE	IDENTIFIER
**Chemicals, peptides, and recombinant proteins**		

DMEM high-glucose	GE Healthcare	Cat# SH30081.01
Bovine calf serum	GE Healthcare	Cat# SH30072.03
L-Glutamine	Wisent	Cat# 609065EL
Penicillin-streptomycin solution	Wisent	Cat# 450201
Nunc 8-well slide (Permanox)	Lab-Tek	Cat# 177445
Phosphate-buffered saline	Wisent	Cat# 311-010-CL
Paraformaldehyde	Thermo Fisher Scientific	Cat# A1131322
Poly-L-Lysine	Sigma-Aldrich	Cat# A-005-M
Glutaraldehyde 2.5% in Sodium Cacodylate Buffer	Electron Microscopy Sciences	Cat# 15960
Diamond Knife (Ultra 35°)	Electron Microscopy Sciences	Cat# 30-SL
Epon-812 or EMbed-812	Electron Microscopy Sciences	Cat# 14900
Whatman qualitative filter paper	GE Life Sciences	Cat# 1001-0155
Sodium cacodylate buffer, 0.2 M, pH 7.4	Electron Microscopy Sciences	Cat# 11650
Calcium chloride solution, Fisher Chemical	Fisher Scientific	Cat# SC101
Osmium tetroxide aqueous solution	Electron Microscopy Sciences	Cat#19100
Uranyl acetate, reagent, A.C.S.	Electron Microscopy Sciences	Cat# 22400
UranyLess	Electron Microscopy Sciences	Cat# 22409
Potassium ferrocyanide aqueous solution	Electron Microscopy Sciences	Cat# 25154-2
Carbon/Formvar covered copper grid	Electron Microscopy Sciences	Cat# FCF400CU50
1.5 mL low protein-binding microtube	SARSTEDT	Cat# 72.703.600
5 nm gold beads	Cytodiagnostics	Cat# CGN5K-5-2
0.05% Trypsin/0.53 mM EDTA	Wisent	Cat# 325-042-CL
Ethanol/ethyl alcohol, pure	Sigma	Cat# 459836
Glutathione beads	Sigma-Aldrich	G4251
Glutathione sepharose bead slurry	GE Healthcare Life Sciences	17075605
Isopropyl-β-D-thiogalactoside (IPTG)	Biobasic	IB0168
Syringe filter 0.22-μm	Mandel Scientific	WYV-SFNY013022NC

**Bacterial and virus strains**		

BL21(DE3) Competent *E. coli* cells	New England Biolabs	Cat # C2527H

**Recombinant DNA**		

GST-α-synuclein plasmid, human	Maneca et al.^3^	N/A
GST-HRV 3C plasmid	Maneca et al.^3^	N/A
pGEX6P1	University of Dundee MRC Protein Phosphorylation and Ubiquitination Unit	Cat# DU30005

**Critical commercial assays**		

Mycoplasma detection kit	Biotool	Cat# B39038
Bradford Assay Kit	Thermo Fisher Scientific	Cat# 23236

**Experimental models: Cell lines**		

U-2 OS	ATCC	Cat# HTB-96

**Software and algorithms**		

ImageJ	Schneider et al.^4^	https://imagej.nih.gov/ij/
Malvern Zetasizer Software	Malvern Panalytical	https://www.malvernpanalytical.com/en/support/product-support/software/Zetasizer-family-software-update-v8-02
GMS 3	Gatan	https://www.gatan.com/products/tem-analysis/gatan-microscopy-suite-software

**Other**		

Tecnai 12 BioTwin 120 kV transmission electron microscope (TEM) or Tecnai G2 Spirit Twin 120 kV Cryo-TEM	FEI	N/A
The Perfect Loop	Science Services	Cat# E70944
Zetasizer Nano S	Malvern Panalytical	https://www.malvernpanalytical.com/en/support/product-support/zetasizer-range/zetasizer-nano-range/zetasizer-nano-s
Digital heating shaking thermomixer	Thermo Fisher Scientific	Cat# 88880027
Bioruptor® Plus sonication device with metallic soundproof box (B01200001) and water cooler (B02010003)	Diagenode	Cat# B01020001

## Materials and equipment

**Table:** U2OS complete growth medium (store at 4°C when not in use)

Reagent	Final concentration	Amount
DMEM high-glucose	N/A	500 mL
Bovine calf serum	10%	56 mL
L-Glutamine	200 μM	5 mL
Penicillin-Streptomycin 10,000 U/mL (100×)	1×	5 mL

## Step-by-step method details

This protocol explains the conjugation of PFFs to nanogold beads while preventing PFFs from aggregating into longer or more aggregated fibrils. Once the nanogold PFFs are generated, the protocol describes the use of EM to visualize their internalization into cells and their subsequent cellular trafficking. This protocol can be used to visualize the cellular trafficking itineraries of numerous proteins. Furthermore, this protocol can be used to examine the internalization of PFFs and trafficking in various cell types, provided that the appropriate changes are made to facilitate cell attachment to the plate (e.g., poly-L-ornithine and Laminin would be used instead of poly-L-lysine for neuronal cultures).

PFFs are conjugated with 5 nm nanogold beads, and a small portion of the conjugated sample is prepared and characterized using EM (steps 1–3: PFFs conjugation and characterization). Provided that the nanogold-labeled PFFs match the ideal criteria (see below for details), cells are plated onto poly-L-lysine treated Nunc 8-well Permanox plates, and nanogold-PFFs are added to the cells (steps 4–8: cell attachment, culturing, and PFFs administration, fixation). Finally, a brief description of the processing of the cell monolayers for EM is provided (steps 9 and 10: processing samples and imaging with EM).

The main advantage of an EM approach to protein internalization is that the specific details involved in internalization (e.g., membrane ruffling and the attachment of proteins to cell membrane) can be visualized directly, rather than using fluorescent microscopy and antibodies or more indirect approaches. We hope that this protocol can be used to elucidate other trafficking pathways for other proteins, specifically those involved in neurodegenerative diseases.

### PFFs conjugation and characterization

**Timing: 1 day**

In this section, we explain the protocol used to conjugate PFFs to 5 nm nanogold beads (Cytodiagnostics). We altered and optimized the standard conjugation protocol provided by the manufacturer (see product sheet on the manufacturer’s website: https://www.cytodiagnostics.com/collections/NHS-Activated-Gold-Nanoparticles).

***Note:*** Since PFFs are very susceptible to aggregation, the nanogold-PFFs need to be administered to cells immediately after conjugation. Prepare the cells such that they are ready to coincide with the conjugation of PFFs to nanogold. Following the administration of PFFs to cells, proceed to prepare grids to characterize the nanogold-PFFs.

***Note:*** all of step 1 should be performed in a tissue culture hood.

1. The conjugation of PFFs with 5 nm nanogold.
  a. Add 48 μL of 5 mg/mL PFFs to 60 μL of the reaction buffer provided with the nanogold conjugation kit.
    i. Thaw reaction buffer for 1 h on ice.
    ii. Retrieve 48 μL of 5 mg/mL PFFs (stored at −80°C) and immediately add 60 μL of ice-cold reaction buffer to the aliquot.
    iii. Incubate for 2 min at room temperature.
    iv. Slowly pipette the mixture up and down.
  b. Add the reaction buffer-PFFs mixture to 1 vial of 5 nm nanogold.
  c. Incubate for 1 h at 18°C.
  d. Add 10 μL of quencher buffer (provided by the manufacturer) to the mixture.
    i. Slowly pipette up and down using a p10 pipette set at 10 μL.
    ii. Avoid creating bubbles in the solution.
  e. Incubate at room temperature for 5 min.

***Note:*** the amount of reaction buffer and quencher buffer used, the amount and concentration of protein used, are all specified by the manufacturer (Cytodiagnostics). The steps above are an adaptation of their protocol for our specific conjugation assay.

***Note:*** for PFFs visualization (labeled with nanogold beads, use a magnification between ×30,000 and ×68,000).

**CRITICAL:** Avoid rapid pipetting of the samples or sudden movements of the experimental microtube as they will aid in protein aggregation.

2. Characterization of conjugated PFFs.
  a. Prepare a diluted sample of PFFs with a 1 mg/mL concentration.
    i. Dilute using ddH_2_O.
  b. Place one drop of diluted PFFs on a carbon/formvar covered copper grid and allow the sample to sit for 10 min at room temperature.
    i. Using a Whatman filter paper, remove the drop from the grid.
  c. Fix the PFFs sample on the grid using 2% PFA (retrieved immediately from storage at 4°C).
    i. Place one drop onto the grid and let the sample sit for 10 min at room temperature.
    ii. Remove PFA drop using filter paper.
  d. Add one drop of ddH_2_O onto the grid for 1 min at room temperature.
    i. Remove with filter paper.
    ii. Repeat once more.
  e. Stain the grid with Uranyless.
    i. Centrifuge 1 mL of Uranlyess at 14,000 × *g*.
    ii. add one drop of the supernatant to the grid and wait for 5 min at room temperature.
  f. Wash grid with ddH_2_O as detailed in **d**.
  g. Remove the drop using a Whatman filter paper.
  h. Let the grid sit overnight on parafilm at room temperature.

**CRITICAL:** place grids in a box/chamber for their storage overnight to avoid the addition of contaminants (such as bacteria, dust, etc.) to the EM grid.

**Figure 2:**
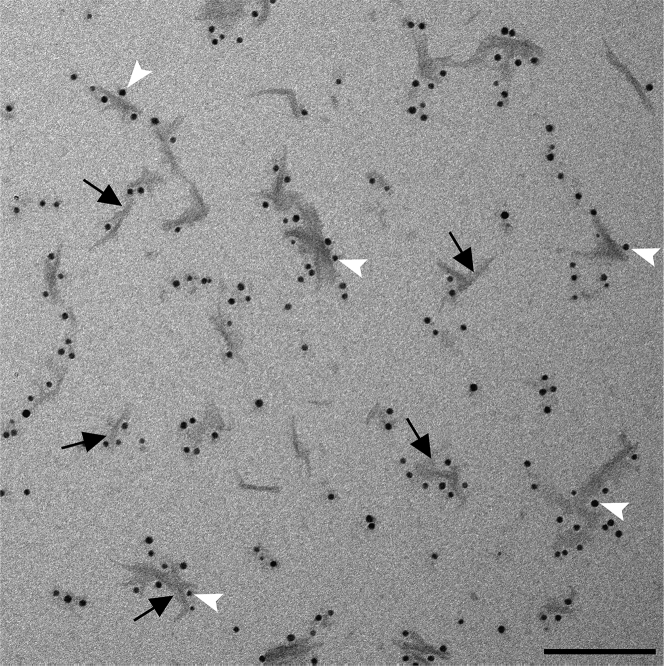
Electron micrograph of nanogold conjugated PFFs Uranyless was used for staining. Arrowheads point to a few examples of conjugated nanogold beads. Arrows indicate optimal examples of nanogold-labeled PFFs: small size and the appropriate amount of conjugation (>1 and <20 beads per PFFs). Scale bar = 100 nm.

3. Visualize using EM to assess and measure the length of fibrils.
  a. See Figure 2 for an example:

Criteria for successful/ideal PFFs conjugation:

1. The majority of PFFs should have a length of less than 100 nm.
2. Most PFFs should be conjugated with at least one nanogold bead.
3. Only 1 in 20 nanogold beads should be unconjugated (free-floating).

### Cell attachment, culturing, and PFFs administration

**Timing: ∼3 days**

Here, we will be culturing and plating cells onto Nunc 8-well Permanox plates previously treated with poly-L-lysine. The protocol described below uses U2OS cells; however, this protocol can be adapted for use with other cell types. We have used the same protocol to examine the internalization of PFFs in primary human astrocytes and glioblastoma cell lines (i.e., U87 and U251).

4. Treat 8-well Permanox plates with poly-L-lysine.
  a. Wash wells with 100% ethanol.
    i. Repeat 3 times.
  b. Wash wells with ddH_2_O.
    i. Repeat 3 times.
  c. Add 200 μL of 0.01% poly-L-lysine (stock concentration and working concentration) to each well.
    i. Incubate wells for 1 h at 37°C.
  d. Wash wells with ddH_2_O.
    i. Repeat 3 times.

**CRITICAL:** For better attachment, allow cells to grow in wells for over 48 h. Allowing cells 48 h between passaging and experimentation will result in healthier cells, which will improve experimental results (i.e., PFFs uptake) and a lower incidence of cells lifting due to fixation.

5. Subculture U2OS cells.
  a. Trypsinize U2OS cells currently in culture, pellet, remove supernatant, resuspend with growth media.
  b. Plate ∼50,000 cells per well.
  c. A total of 150–200 μL of media per well is optimal.
  d. Incubate for 48–72 h.

***Note:*** Before the experiment, use the mycoplasma kit to test samples for mycoplasma infection. The presence of mycoplasma could have effects on cellular processes, altering your experimental results.

6. Add 5 μL of conjugated PFFs to each well (assuming a final concentration of 2 mg/mL of conjugated PFFs).
  a. Following the addition of conjugated PFFs to each well, incubate cells for 30 min at 37°C.
  b. Remove cell media.
7. The following step is optional as it details our exclusion assay involving the use of trypsin to wash off extracellular PFFs.
  a. Place cells on ice and administer 200 μL of ice-cold trypsin (0.05% Trypsin/EDTA 0.53 mM) to each well.
  b. Incubate for 60–90 s on ice.
    i. exposure time may need to be adjusted to prevent the lifting of cells.
  c. remove trypsin and wash cells with 200 μL of ice-cold PBS.
  d. wash cells gently with PBS.
8. Sample fixation.
  a. fix cells with 2.5% glutaraldehyde (in sodium cacodylate buffer) supplemented with 2 mM Calcium chloride (CaCl_2_).
    i. CaCl_2_ is used to aid in membrane preservation during fixation. The concentration might need to be optimized for specific cell types.
    ii. Fix cells at 4°C overnight (a minimum of 2 h of fixation is recommended at 4°C).
  b. Wash cells with 0.1 mM sodium cacodylate buffer (dilute stock solution using ddH_2_O).
    i. Remove fixative solution.
    ii. Add 200 μL of sodium cacodylate buffer to each well.
    iii. Aspirate the buffer.
    iv. Replace with 200 μL of sodium cacodylate buffer.

***Note:*** a more detailed description of handling the samples will be provided in step 9.

### Processing samples and imaging with EM

**Timing: 2–3 days**

Here, we discuss the steps to process the monolayer samples for EM imaging. This should be conducted following overnight fixation of samples in 2.5% glutaraldehyde.

***Note:*** post-fixation and block staining should be done in a fume hood.

9. Processing samples for EM.
  a. Wash out glutaraldehyde fixative.
    i. Remove fixative.
    ii. Add 300 μL of 0.1 M sodium cacodylate buffer.
    iii. Incubate for 10 min at room temperature.
    iv. Repeat i-iii twice.
  b. Post-fix samples using 1% osmium tetroxide and 1.5% potassium ferrocyanide (diluted in ddH_2_O).
    i. Add potassium ferrocyanide powder in ddH_2_O.
    ii. Add the appropriate amount of osmium tetroxide to the mixture (based on the concentration used).
    iii. Add 200 μL of the mixture to each well.
    iv. Incubate at 4°C for 1–2 h.
  c. Wash out the post-fixative solution.
    i. Wash cells with ddH_2_O.
    ii. Incubate for 10 min at room temperature.
    iii. Repeat i and ii twice.
  d. Gradual dehydration of samples.
    i. Aspirate ddH_2_O.
    ii. Add 30% ethanol/ddH_2_O (dilute using pure ethanol).
    iii. Incubate at room temperature for 10 min.
    iv. Repeat i-iii using 50%, 60%, and 70% ethanol solutions.
  e. Block staining with uranyl acetate.
    i. Prepare a solution of 2% uranyl acetate in 70% ethanol.
    ii. Add 200 μL of staining solution to each well (200 μL volume should be used for all the following steps).
    iii. Incubate at 4°C for 2 h
  f. Wash out the staining solution.
    i. Aspirate samples.
    ii. Wash samples twice with 70% ethanol.
  g. Continue sample dehydration.
    i. Aspirate wells.
    ii. Add 80% ethanol solution to each well.
    iii. Incubate for 10 min at room temperature.
    iv. Repeat i-iii once more, and remove the solution.
    v. Add 90% ethanol solution to each well.
    vi. Incubate for 15 min at room temperature.
    vii. Repeat v-vi once more, and remove the solution.
    viii. Add 100% ethanol solution to each well.
    ix. Incubate for 15 min at room temperature.
    x. Remove solution.
    xi. Repeat viii-x twice more.
  h. Infiltration with Epon-812.
    i. Add a 50% solution of Epon-812 (diluted in 100% ethanol) to each well.
    ii. Incubate at room temperature for 1 h.
    iii. Aspirate wells.
    iv. Add a 75% solution of Epon-812/ethanol to each well.
    v. Incubate at room temperature for 1 h.
    vi. Aspirate wells.
    vii. Add a 100% solution of Epon-812 to each well.
    viii. Incubate at room temperature for 1 h.
    ix. Aspirate wells.
    x. Add a 100% solution of Epon-812 to each well.
  i. Incubate samples with Epon-812 at 60°C for 48 h.
    i. Allow samples to cool at room temperature overnight before sectioning.
  j. Trim and section prepared blocks.
    i. Trim the block to a small area of interest (use a light microscope to find regions of interest).
    ii. Use a diamond knife to section samples (Ultra 35°) – samples will end up floating in the ddH_2_O-filled boat of the diamond knife where they can be collected.
    iii. Place one section onto a grid using the Perfect Loop tool.
    iv. Place the grid onto a Whatman filter paper and allow it to dry.
    ***Note:*** ideally, each section should be 70–100 nm thick.
  k. Stain grids with uranyl acetate and lead citrate (concentration is based on tissue and staining preferences).

***Note:*** our grid staining protocol is based on a previously published paper by Santhana Raj et al.^4^

***Note:*** EMbed 812 is a suitable alternative to Epon-812.

**CRITICAL:** Do not let samples dry out. Ensure that they do not go without a covering of liquid for more than 10 s.

10. Image grids using a transmission EM at ×9300 to ×18500 magnification (allowing imaging of the fibrils along with cellular structures).

***Note:*** Electron micrographs can be viewed and analyzed with ImageJ or GMS software, both freely available at the links mentioned in the key resources table.

## Expected outcomes

**Figure 3:**
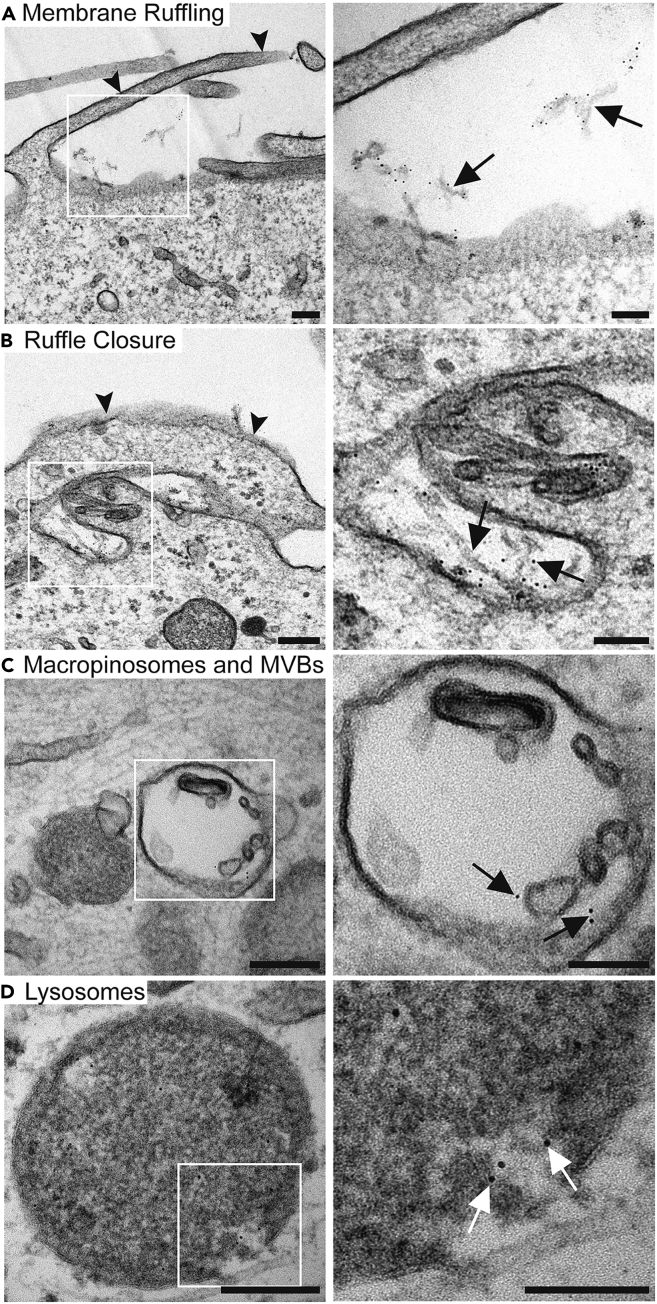
Internalization of nanogold-PFFs visualized using EM (A–D) Images show the internalization of PFFs in U2OS cells in 4 stages. Nanogold PFFs (arrows) are seen at the cell surface, being engulfed by membrane ruffles (arrowhead). They are then trafficked to macropinosomes, early multivesicular bodies (MVBs), and lysosomes. Scale bar = 200 nm, and 100 nm for insets.

One would expect to observe conjugated PFFs with mostly small, preformed fibrils (<100 nm) and not large, aggregated fibril structures. We expect that the internalization and the trafficking of nanogold-PFFs can be easily spotted in cells when viewing sections using EM. In Figure 2, one can see an example of what a typical fibril sample should look like: mostly small, preformed fibrils, conjugated by 2–8 nanogold particles per PFFs. In Figure 3, an example of the cellular internalization of PFFs using EM has been shown.

***Note:*** as mentioned above, the objective is to have a very small number of free nanogold beads that have not been conjugated with PFFs.

## Limitations

One major limitation of this protocol is that there is no specific ratio between PFFs and the number of conjugated nanogold particles. This makes quantification of the results difficult as the number of nanogold particles does not directly imply the size of the fibrils or the number of fibrils when imaging intracellular PFFs trafficking.

The other limitation is the inability to control the size of the resulting conjugated fibrils. Although our protocol yields many PFFs with a length that is less than 100 nm, the appearance of larger fibrils cannot be avoided. We believe that this issue is largely moot in quick endocytic experiments, where administered nanogold-PFFs are in contact with cells for 30 min or less. It remains that our protocol does not allow for PFFs size-related experiments to occur (i.e., 50 nm PFFs internalization vs 75 nm PFFs internalization).

## Troubleshooting

While certain outcomes are unavoidable using our protocol, such as the presence of a small number of free-floating nanogold particles, and the formation of large fibrils conjugated with nanogold, experimental conditions can be optimized to reduce the likelihood of such events occurring. Here we address some of the common issues that can be addressed. As a guiding principle, it is okay to have unconjugated PFFs if almost all nanogold beads are attached to PFFs (i.e., unconjugated PFFs are acceptable but unconjugated nanogold beads are not).

### Problem 1

Presence of unconjugated nanogold particles.

**Figure 4:**
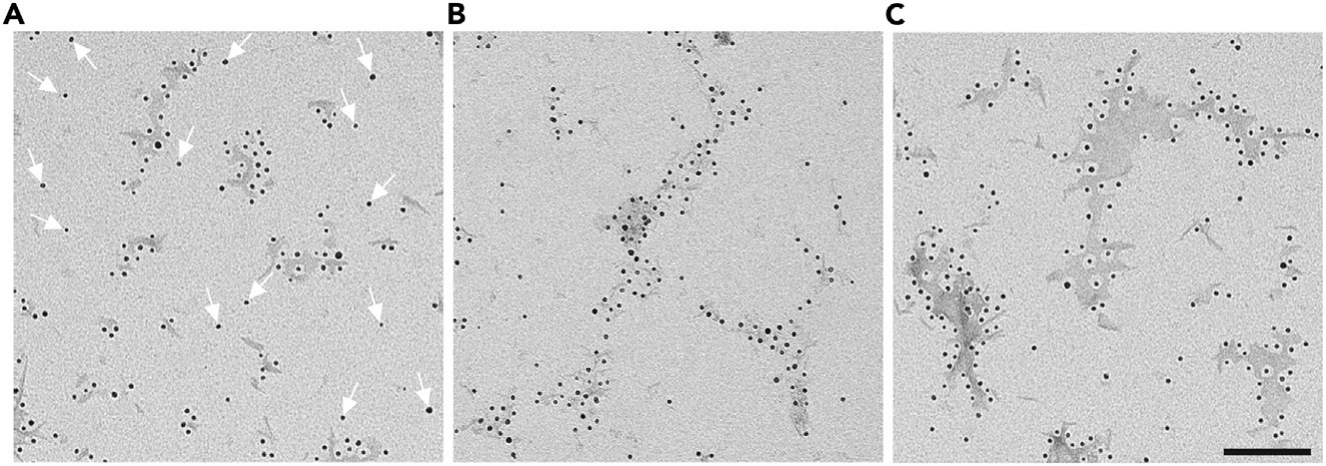
Problematic outcomes of PFFs conjugation (A) An example of many free-floating unconjugated nanogold beads (arrows). (B) An example of PFFs over-conjugation with nanogold particles. (C) An example of PFFs conjugation that results in large PFFs aggregates spanning several hundred microns. Scale bar = 100 nm.

Our guideline for using a sample is that only 1 out of 20 nanogold particles can be unconjugated when characterizing a conjugated sample. Should free gold particles be more frequent (Figure 4A), the sample should be discarded, and the conjugation process should be optimized. Below, we outline some ways in which this problem can be alleviated.

### Potential solution

Manipulate the temperature or duration of the conjugation experiment.

Instead of incubating the mixture of reaction buffer, nanogold, and PFFs at 18°C as suggested by our protocol, try incubating the mixture at a higher temperature (e.g., 21°C/room temperature). This will ensure that more covalent bonds occur between the PFFs and nanogold beads.

Increased conjugation of nanogold beads with PFFs can also be achieved by increasing the incubation time from 1 h to 1.5 or 2 h. This enables the ability to keep the incubation temperature at 18°C which results in a less aggregated product.

### Problem 2

Over-conjugation of PFFs.

While successful conjugation of PFFs with multiple nanogold beads is the desired outcome, over-conjugation of fibrils with nanogold beads can also occur (Figure 4B). This is not optimal, as the increased size and weight of the resulting conjugated protein might affect its internalization and delay or even prevent its uptake by cells. Below we describe a solution that effectively eliminates this issue.

### Potential solution

Increase PFFs concentration.

As the conjugation experiment is a chemical reaction that results in the formation of covalent bonds between PFFs and nanogold, stoichiometry, or more simply, the amount of each reactant is important. By increasing the number of PFFs added, through the increase of concentration and not volume, you can decrease the likelihood of over-conjugation of nanogold particles onto PFFs. A concentration range of 1–5 mg/mL is desirable when conducting the conjugation experiment. Refer to the manufacturer’s protocol regarding the desirable concentration for different sizes of nanogold beads.

### Problem 3

Presence of large fibril aggregates: Most common.

The most common, and least favorable outcome is the presence of conjugated PFFs with lengths over 100 nm. Although a low number of such fibrils will occur, samples that mostly contain these large structures must be discarded (Figure 4C), since the conjugated proteins can no longer be referred to as PFFs, and hence, may utilize a different pathway for entry and trafficking into cells.

### Potential solution

Manipulate experimental conditions and utilize proper handling of PFFs.

Prior to conjugation, characterize the PFFs batch by aliquoting and visualizing the unconjugated sample using EM. If the presence of large fibrils is frequent, do not conjugate the batch as the issue with PFFs length will only be exacerbated during conjugation.

In addition, avoid the following behaviors when conjugating nanogold beads onto PFFs:

• rapid movement of the sample.
• holding the tube with PFFs between one’s fingers.
  ○ the transfer of body temperature to the tube can result in accelerating the aggregation process.
• rapid pipetting up and down.
• repeated freeze and thaw.

Another solution to reduce the aggregation of conjugated PFFs is to allow the experiment to occur at a lower temperature. The correct temperature (e.g., room temperature, 4°C, 37°C, etc.) for your experiment will therefore be a balance between decreased aggregation and decreasing the likelihood of nanogold conjugation onto PFFs.

### Problem 4

Spotting of nanogold-PFFs in lysosomes.

Lysosomes are electron-lucent organelles when observed using EM, which makes it difficult to spot nanogold-conjugated proteins in their lumen. Although this issue can often be resolved by adjusting image contrast, sometimes a change in protocol is necessary for clear viewing of lysosomal contents.

### Potential solution

No grid staining.

In our protocol, the samples are stained with uranyl acetate both prior to embedding (*en bloc*) and after embedding (grid staining). To lower the overall staining of the samples, and have a better view of the lysosomal lumen, avoid grid staining with uranyl acetate and lead citrate. This will result in samples with much less staining, and hence, a better look into the lysosomal lumen.

### Problem 5

Uranyl acetate-related artifacts.

Uranyl acetate staining can have potential drawbacks since its exposure to light results in its precipitation. Stock solutions of uranyl acetate are also prone to precipitation with age. The result will be large, dark artifacts in EM samples, which will result in suboptimal images.

### Potential solution

Regular filtration and centrifugation of uranyl acetate before use.

To avoid uranyl acetate-related artifacts, first centrifuge an aliquot of uranyl acetate to pellet aggregate chunks. Collect the supernatant and run it through a 0.22-μm filter. Uranyl acetate can now be used for sample staining.

## Resource availability

### Lead contact

Further information and requests for resources and reagents should be directed to and will be fulfilled by the lead contact, Armin Bayati, armin.bayati@mail.mcgill.ca, or Peter McPherson, peter.mcpherson@mcgill.ca.

### Materials availability

No new data was generated in this study.

## Data Availability

Protocol did not generate any quantitative data. Original images are available upon request from the lead contact.
